# The association between shift work, occupational physical activity and cardiovascular-kidney-metabolic syndrome: a cohort study in China

**DOI:** 10.3389/fpubh.2026.1831468

**Published:** 2026-06-03

**Authors:** Xiangran Zhang, Wanyu Wang, Haixia Lu, Changxue Huang, Yuxin Jin, Manli Xiang, Cainan Sun, Di Li, Li Chen, Xiujuan Wu, Hongzhan Sun, Yulong Lian, Dajun Zhang

**Affiliations:** 1 Department of Epidemiology and Medical Statistics, School of Public Health, Nantong University, Nantong, China; 2 Karamay Central Hospital, Karamay, Xinjiang Uygur Autonomous Region, China; 3 Karamay Center Disease Control and Prevention, Karamay, Xinjiang Uygur Autonomous Region, China

**Keywords:** CKM syndrome, cohort study, joint effect, occupational physical activity, shift work

## Abstract

**Background:**

The relationship between shift work, occupational physical activity (OPA), and CKM syndrome remains unclear. The present study aimed to investigate the independent and joint associations of these two factors with CKM syndrome.

**Methods:**

This study conducted a baseline survey in 2013 and a follow-up in 2023, with a total of 690 workers finally enrolled. CKM syndrome was diagnosed according to the American Heart Association (AHA) criteria. Participants were classified into fixed day shifts and shift work, and OPA into light/moderate and heavy/very heavy groups. Poisson regression was adopted to explore the associations of shift work and OPA with CKM syndrome, as well as their joint effects.

**Result:**

Compared with fixed day shifts, shift work was associated with CKM syndrome (RR = 1.568, 95% CI: 1.328–1.852); two-shift (RR = 1.677, 95% CI: 1.391–2.022), three-shift (RR = 1.502, 95% CI: 1.223–1.844), four-shift (RR = 1.529, 95% CI: 1.235–1.892). The risk of CKM syndrome showed a dose–response relationship with cumulative night shift exposure. Workers with heavy or very heavy OPA had a higher risk of CKM syndrome (RR = 1.432, 95% CI: 1.239–1.654) than those with light or moderate OPA.

**Conclusion:**

Both shift work and heavy or very heavy OPA increase the risk of CKM syndrome. The risk rises with increasing cumulative night shift exposure. Workers with both shift work and heavy or very heavy OPA have the highest risk. Therefore, early screening for CKM syndrome should be strengthened among shift workers and those with heavy OPA.

## Introduction

Cardiovascular kidney-metabolic (CKM) syndrome is a new medical concept proposed by the American Heart Association (AHA) in 2023. It is a systemic disease caused by the pathophysiological interaction among metabolic risk factors, chronic kidney disease (CKD), and the cardiovascular system (1). Approximately 90% of American adults and 76.4% of Chinese adults meet the diagnostic criteria for CKM syndrome (2, 3). It can lead to multiorgan dysfunction and a high incidence of adverse cardiovascular events, imposing a heavy disease burden on society (4).

With economic development, shift work has become a common occupational risk factor in industrialized countries, accounting for more than 20% of the total labor force (5). Shift work is generally defined as regular rotational work outside normal daytime hours (17:00–08:00 the next day) (6). Numerous studies have indicated that shift work is associated with the components of CKM syndrome, including obesity (7), metabolic syndrome (Mets) (8), cardiovascular disease (CVD) (9), and CKD (10). Xi et al. (11) reported that each 5-year increase in shift work duration was associated with a 7% higher risk of cardiovascular disease. Ding et al. (12) found that evening or night work schedules were associated with higher risks of coronary heart disease and angina. Li et al. (13) found that shift work was related to an increased risk of diabetes, with a significant linear dose–response relationship between shift work duration and diabetes risk. Tran et al.’s (14) study examined the association between shift work and CKM conditions, but the definition of CKM syndrome used in this study was inaccurate. No study has investigated the association between shift work and CKM syndrome defined by the AHA criteria.

To date, the relationship between occupational physical activity (OPA) and CKM syndrome remains inconclusive. Several studies have investigated the association between OPA and the individual components of CKM syndrome, but the results are inconsistent. Li et al. (15) reported that high-intensity occupational physical activity increased the risk of cardiovascular disease by 10–30%, whereas Kazemi et al. (16) found no significant association between heavy OPA and the incidence of cardiovascular disease, congenital heart disease, stroke, or atrial fibrillation. Panizo González et al. (17) reported that extremely high-intensity exercise can induce renal tubular injury. For type 2 diabetes mellitus, Matthews et al. (18) reported that heavy OPA may increase the risk; but other studies showed conflicting results. Swedish cohort study (19) showed no significant association between OPA and the risk of diabetes onset, whereas a Finnish study (20) reported that moderate and high levels of OPA reduce the risk of type 2 diabetes. Seo et al. (21) found no significant association between OPA and MetS. Therefore, this study aims to further investigate this issue.

Shift work and OPA frequently coexist in industrial populations, especially in resource extraction and manufacturing sectors, where a large proportion of frontline shift workers are engaged in heavy manual labor. A study by Loef et al. revealed that workers engaged in physical labor accounted for 37.4% of all shift workers (22), while Loprinzi (23) reported that moderate and vigorous OPA performed by shift workers constituted 59 and 70% of their total physical activity, respectively. In the petroleum industry, for instance, rotating shifts are commonly combined with prolonged, strenuous physical tasks, creating dual exposure that jointly affects cardiometabolic and renal health. Biologically, the two factors contribute to CKM syndrome through distinct but complementary pathways: shift work-induced circadian disruption suppresses melatonin secretion, dysregulates cortisol rhythm, impairs insulin sensitivity, and triggers metabolic abnormalities, thereby promoting CKM syndrome development (24). And, heavy or very heavy OPA leads to chronic mechanical strain, recurrent blood pressure elevations, sustained systemic inflammation, and renal hemodynamic stress, collectively promoting endothelial injury, renal impairment, and metabolic dysregulation (25, 26). Given their high co-occurrence in working populations and independent yet synergistic biological mechanisms, investigating their individual and combined risks for CKM syndrome is scientifically warranted.

The study proposes the following hypotheses: (1) shift work increases the risk of CKM syndrome in workers; (2) heavy or very heavy OPA increases the risk of developing CKM syndrome; (3) combined exposure to shift work and heavy/very heavy OPA is associated with a higher risk of CKM syndrome. Ultimately, this study aims to explore the impact of shift patterns and OPA on the risk of CKM syndrome, providing a scientific basis for reducing the incidence of CKM syndrome among workers.

## Methods

### Study participants

This study is based on the Occupational Health Study of Petroleum Industry Workers in Xinjiang Uygur Autonomous Region, China (OHSPIW) (27). In this study, we first stratified by enterprise scale and randomly selected 4 large, 4 medium, and 4 small enterprises. Further random sampling was conducted: 100 individuals from each large enterprise (>500 employees), 70 from each medium enterprise (300–500 employees), and 30 from each small enterprise (<300 employees), with a total of 800 participants. These participants underwent health checks at the Karamay City Center for Disease Control and Prevention in 2013 and completed a questionnaire containing basic information. The main contents of the questionnaire include basic demographic information, lifestyle, personal past and personal career history, etc. The main contents of the physical examination include measuring height, weight, waist circumference, blood pressure, blood routine, fasting blood glucose, urine routine, etc. Eligible participants for the study were employees of the Karamay Petroleum Administration Bureau and Petrochemical Company who had worked in their positions for 1 year and were aged 20–60 years.

The required sample size was calculated using the formula for comparing two proportions in a cohort study:


$$n=\frac{z_{\alpha }\sqrt{2\bar{\mathrm{pq}}}+\mathrm{Z\beta}\sqrt{{\left(p_{0}q_{0}+p_{1}q_{1}\right)}^{2}}}{{\left(p_{1}-p_{0}\right)}^{2}}$$

Since the incidence rate of CKM syndrome is currently unknown, we used the incidence rate of MetS—a key component of CKM syndrome—as a reference. Based on the study by De Bacquer et al. (8), the annual incidence rate of MetS was 60.6 per 1,000 person-years among shift workers and 37.2 per 1,000 person-years among day workers, with RR of 1.77. With *α* = 0.05 and *β* = 0.20, the required sample size per group was calculated to be 178. After accounting for an expected 15% loss to follow-up, a total of 410 participants were needed for the two groups, which is satisfied by the sample size of the present study.

*Post hoc* power analysis was performed using G*Power 3.1.9.7. With a two-sided *α* of 0.05, assuming a CKM syndrome prevalence of 64.3% in the exposed group and 42.0% in the control group, and a total sample size of 690 (409 in the exposed group and 281 in the control group), the achieved power of this study was 0.99995, which is well above the conventional threshold of 0.80, indicating that the study has excellent statistical power to detect the hypothesized association.

**Figure 1:**
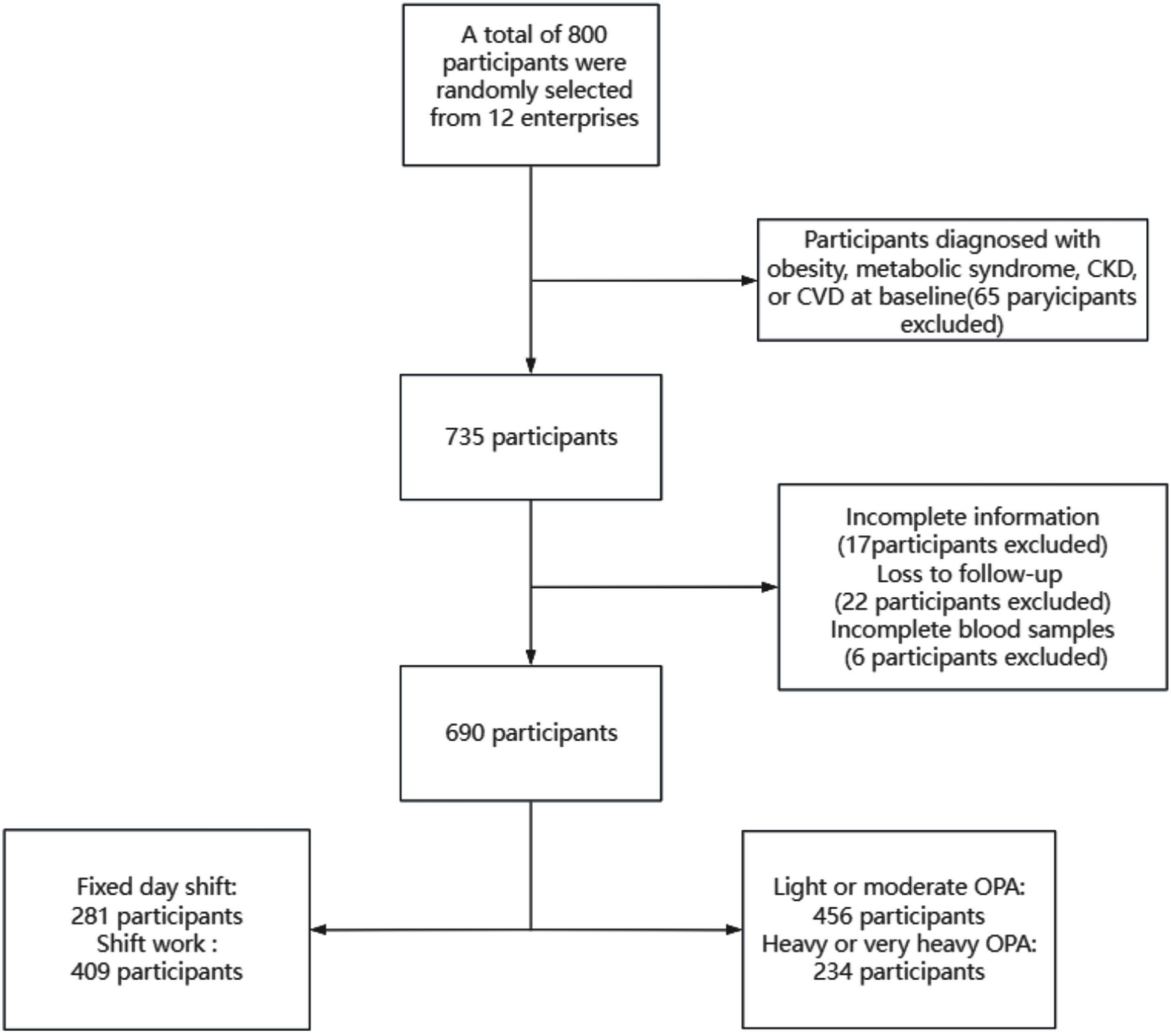
Flow chart of sample selection.

Moreover, participants meeting the following criteria were excluded: (1) participants diagnosed with obesity, metabolic syndrome, CKD, or CVD at baseline.; (2) those with incomplete questionnaire information; (3) those who were transferred from their positions, did not participate in the health check, or were lost to follow-up; 4) those from whom blood samples were not collected or whose blood samples were unqualified. Follow-up surveys were conducted with the participants in 2023, with the same content as the baseline survey. A total of 110 participants were excluded, and 690 participants were included in the study. The baseline characteristics of the included and excluded participants are presented in Supplementary Table S1, and the flow chart of sample selection is shown in Figure 1.

This study was approved by the Nantong University Ethics Committee (2013-L073). All participants signed an informed consent form after receiving information about the study.

### The diagnostic criteria for CKM syndrome

According to the definition of CKM syndrome in the American Heart Association (AHA) statement for CKM syndrome (1), the syndrome is categorized into five stages: Stage 0 refers to individuals with no CKM risk factors. Stage 1 is defined by the presence of excessive or dysfunctional adipose tissue, including overweight/obesity, abdominal obesity, or impaired glucose tolerance. Stage 2 involves the presence of metabolic risk factors (such as hypertriglyceridemia, hypertension, diabetes, and metabolic syndrome) along with CKD. Stage 3 is CKM Syndrome complicated by subclinical CVD. Stage 4 is CKM Syndrome complicated by clinical CVD, including conditions, such as coronary heart disease, heart failure, stroke, peripheral arterial disease, and atrial fibrillation. In this study, CKM syndrome stage 0 was defined as the absence of CKM syndrome, while stages 1–4 were defined as the presence of CKM syndrome.

### Shift work

This study collected baseline information on shift work through questionnaire. In accordance with the Convention Concerning Night Work (28) of the International Labour Organization (ILO), fixed day shifts are defined as work hours from 08:00 to 17:00. Employees working night shifts were classified as shift workers and grouped into two shifts, three shifts, and four shifts: “Two shifts” consisted of two 12-h shifts with two worker groups alternating weekly; “Three shifts” included two 12-h shifts with three worker groups alternating weekly and one group resting; and “Four shifts” comprised three 8-h shifts (morning, middle, and evening) with four worker groups rotating and one group resting.

The monthly number of night shifts was determined based on the reported shift patterns: 13 nights per month for two shifts, 8 nights per month for three shifts, and 4 nights per month for four shifts. The cumulative number of night shifts was calculated by multiplying the monthly night shifts by 11 months and then by the length of employment. Participants were categorized into no exposure (0 nights), low exposure (1–220 nights), medium exposure (221–660 nights), and high exposure (>660 nights) (29).

### Occupational physical activity

In this study, the intensity of the OPA was determined in accordance with studies on the classification of physical work intensity for petroleum fieldwork (30). Specifically, male oil production workers, male oil transportation workers, and downhole fracturing workers were classified as Level I (light OPA); female oil transportation workers and geophysical drilling workers were classified as Level II (moderate OPA); all other patterns of work were classified as Level III or above (heavy OPA); and geophysical wireline workers had the highest intensity, reaching Level IV (very heavy OPA). Previous studies have shown that workers with high or no OPA have a higher CVD mortality rate compared to those with low OPA (31). Low and moderate OPA appear to have beneficial effects (25), therefore, we divided the OPA into two groups: “light or moderate occupational physical work” and “heavy or very heavy occupational physical work”. In this study, OPA was limited to work tasks and occupational activity patterns during working hours and at the workplace, and did not include physical activities during leisure time.

### Evaluation of covariates

We used a self-filling questionnaire to collect covariates, which mainly included the following contents: basic demographic characteristics (age, gender, ethnicity, marital status, education level), occupational history (job type, average monthly income, length of service), and risk factors related to CKM syndrome (smoking, drinking, leisure-time physical activity, dietary status, and family history of hypertension and diabetes). Covariates were collected based on baseline data.

The main categorical variables are defined as follows: (1) Age (≤39 years, 40–49 years, ≥50 years); (2) Gender (male, female); (3) Ethnicity (Han nationality, other ethnicities); (4) Marital status [unmarried, married, other (divorced, widowed, remarried, unknown)]; (5) Educational level (senior high school or below, junior college, bachelor’s degree or above); (6) Job type (drilling worker, driller, well testing worker, oil production worker, downhole operation (fracturing) worker, oil transportation worker, operator, other (oil refining, mud logging, oil refining, well logging, unknown)); (7) Average monthly income (≤2,999 yuan, 3,000–4,999 yuan, ≥5,000 yuan); (8) Length of service (<5 years, 5–15 years, ≥15 years); (9) Smoking status was divided into “regular smoking (≥1 cigarette/day)”, “occasional smoking (<1 cigarette/day)”, “quit smoking,” and “non-smoking” based on whether the individual smoked at least one cigarette per day for more than half a year (32); (10) Weekly alcohol consumption was quantified in grams (g) including beer, red wine, liquor, rice wine, etc., and alcohol drinking was categorized into “regular drinking (≥8 g/day)”, “occasional drinking (<8 g/day)”, “quit drinking”, and “non-drinking” (33); (11) Leisure-time physical activity specifically refers to physical activities conducted during free time (i.e., outside working hours) such as those related to entertainment, travel, and family, and based on personal interests and needs (e.g., walking, gardening, sports, exercise, dancing, etc.). In this study, the participants were divided into four groups: “no physical activity”, “<3 times/week”, “≥3 times/week”, and “irregular physical activity”; (12) The DASH (Dietary Approaches to Stop Hypertension) diet adherence score was divided into 3 groups from low to high using the tertile method (≤22 points, 23–26 points, ≥27 points) (34); (13) Family history of hypertension (yes, no); (14) Family history of diabetes (yes, no).

### Statistical analyses

Statistical analyses used IBM SPSS Statistics 26.0 and R 4.4.1. Categorical variables were described as frequency (percentage) [*n* (%)], and Pearson’s *χ*^2^ test or Fisher’s exact test was used. Poisson regression models were used to analyze the association strength of shift work, OPA with CKM syndrome among workers. Model 1 was unadjusted; Model 2 was adjusted for gender, age, ethnicity, marital status, average monthly income, and educational level; Model 3 was further adjusted for length of service, smoking status, drinking status, leisure-time physical activity, DASH score, family history of hypertension, and family history of diabetes. All hypothesis tests were two-tailed, with a significance level of *α* = 0.05.

## Results

### General demographic characteristics

A total of 690 participants were ultimately included in this study, whom 347 were males (50.3%) and 343 were females (49.7%). Specifically, 281 participants (40.7%) engaged in fixed day shift, whereas 409 participants (59.3%) were involved in shift work. The number of cases with CKM syndrome was 364, with an incidence rate of 52.8%. Table 1 presents the general demographic characteristics of workers on fixed day shift and shift work. Among the factors analyzed, there were statistically significant differences between shift work status and average monthly income, smoking status, leisure-time physical activity, dietary habits, family history of diabetes (all *p* < 0.05). In addition, factors such as gender, age, and ethnicity were not significantly different across different shift work patterns (all *p* > 0.05).

**Table 1:** General demographic characteristics.

Variable	Overall total population	Fixed day shift *N* = 281 (*n*, %)	Shift work *N* = 409 (*n*, %)	*p*
Gender				0.149
Male	347	132 (47.0)	215 (52.6)	
Female	343	149 (53.0)	194 (47.4)	
Age (years)				0.833
≤39	212	89 (31.7)	123 (30.1)	
40–49	304	120 (42.7)	184 (45.0)	
≥50	174	72 (25.6)	102 (24.9)	
Ethnicity				0.874
Han nationality	662	270 (96.1)	392 (95.8)	
Other ethnicities	28	11 (3.9)	17 (4.2)	
Marital status				0.398
Unmarried	31	9 (3.2)	22 (5.4)	
Married	615	254 (90.4)	361 (88.3)	
Other	44	18 (6.4)	26 (6.4)	
Educational level				0.699
Senior high school or below	89	39 (13.9)	50 (12.2)	
Junior college	527	210 (74.7)	317 (77.5)	
Bachelor’s degree or above	74	32 (11.4)	42 (10.3)	
Job type				0.130
Drilling worker	150	58 (20.6)	92 (22.5)	
Driller	39	17 (6.0)	22 (5.4)	
Well testing worker	58	22 (7.8)	36 (8.8)	
Oil production worker	94	49 (17.4)	45 (11.0)	
Downhole operation worker	112	34 (12.1)	78 (19.1)	
Oil transportation worker	118	49 (17.4)	69 (16.9)	
Operator	76	34 (12.1)	42 (10.3)	
Other	43	18 (6.4)	25 (6.1)	
Average monthly income (yuan)				0.012
≤2,999	19	6 (2.1)	13 (3.2)	
3,000–4,999	615	242 (86.1)	373 (91.2)	
≥5,000	56	33 (11.7)	23 (5.6)	
Length of service (years)				0.806
≤9	82	31 (11.0)	51 (12.5)	
10–19	195	82 (29.2)	113 (27.6)	
≥20	413	168 (59.8)	245 (59.9)	
Smoking status				<0.001
Non-smoking	518	197 (70.1)	321 (78.5)	
Regular smoking	62	13 (4.6)	49 (12.0)	
Occasional smoking	83	56 (19.9)	27 (6.6)	
Quit smoking	27	15 (5.3)	12 (2.9)	
Drinking status				0.160
Non-drinking	331	133 (47.3)	198 (48.4)	
Regular drinking	193	70 (24.9)	123 (30.1)	
Occasional drinking	158	73 (26.0)	85 (20.8)	
Quit-drinking	8	5 (1.8)	3 (0.7)	
Leisure-time physical activity				<0.001
No physical activity”	79	32 (11.4)	47 (11.5)	
<3 times/week	269	72 (25.6)	197 (48.2)	
≥3 times/week	115	77 (27.4)	38 (9.3)	
Irregular physical activity”	227	100 (35.6)	127 (31.1)	
DASH score (points)				<0.001
≤22	188	80 (28.5)	108 (26.4)	
23–26	282	95 (33.8)	187 (45.7)	
≥27	220	106 (37.7)	114 (27.9)	
Family history of hypertension				0.228
No	553	219 (77.9)	334 (81.7)	
Yes	137	62 (22.1)	75 (18.3)	
Family history of diabetes				<0.001
No	437	137 (48.8)	300 (73.3)	
Yes	253	144 (51.2)	109 (26.7)	

### Analysis of the association between shift work and the incidence of CKM syndrome

As shown in Table 2, in Model 3, shift workers had an increased risk of CKM syndrome compared with fixed day shift workers (RR = 1.568, 95% CI: 1.328–1.852). Given no stage 3 and only two stage 4 CKM cases, stages 2–4 were combined for analysis. Shift workers had a higher risk of stage 1 CKM (RR = 2.386, 95% CI: 1.642–3.466) and stages 2–4 CKM (RR = 1.670, 95% CI: 1.342–2.080) than fixed day shift workers.

**Table 2:** Association between shift work and the incidence of CKM syndrome.

Shift work status	Case/total (incidence)	Model 1		Model 2		Model 3	
		RR (95% CI)	*p*	RR (95% CI)	*p*	RR (95% CI)	*p*
CKM syndrome							
Fixed day shift	118/281 (42.0)	1.000	—	1.000	—	1.000	—
Shift work	263/409 (64.3)	1.432 (1.222–1.678)	<0.001	1.420 (1.214–1.661)	<0.001	1.568 (1.328–1.852)	<0.001
CKM syndrome stage 1							
Fixed day shift	36/281 (12.8)	1.000	—	1.000	—	1.000	
Shift work	92/409 (22.5)	1.994 (1.423–2.796)	<0.001	2.030 (1.440–2.863)	<0.001	2.386 (1.642–3.466)	<0.001
CKM syndrome stages 2–4							
Fixed day shift	82/281 (29.2)	1.000	—	1.000	—	1.000	
Shift work	154/409 (37.7)	1.451 (1.177–1.790)	<0.001	1.000 1.444 (1.174–1.776)	<0.001	1.670 (1.342–2.080)	<0.001

Model 1: no adjustment for confounding factors. Model 2: adjusted for gender, age, ethnicity, marital status, average monthly income, and educational level. Model 3: adjusted for gender, age, ethnicity, marital status, average monthly income, educational level, length of service, smoking status, drinking status, leisure-time physical activity, DASH score, family history of hypertension, and family history of diabetes.

Table 3 shows that the risk of CKM syndrome increases significantly with the number of night shifts. Compared with fixed day shifts, workers with more than 660 night shifts have the most pronounced increase in risk, with an RR of 1.687 (95% CI: 1.412–2.015) in the fully adjusted Model 3.

**Table 3:** Association between night shift frequency and CKM syndrome.

Shift work patterns	Case/total (incidence)	Model 1		Model 2		Model 3		*p*-for tend
		RR (95% CI)	*p*	RR (95% CI)	*p*	RR (95% CI)	*p*	
Fixed day shift	118/281 (42.0)	1.000	—	1.000	—	1.000	—	<0.001
1–220 nights	22/48 (45.83)	1.091 (0.779–1.529)	0.611	1.098 (0.797–1.514)	0.567	1.139 (0.805–1.612)	0.463	
220–660 nights	42/72 (58.3)	1.389 (1.094–1.764)	0.007	1.358 (1.073–1.718)	0.011	1.447 (1.130–1.854)	0.003	
>660 nights	182/289 (63.0)	1.500 (1.274–1.766)	<0.001	1.490 (1.264–1.757)	<0.001	1.687 (1.412–2.015)	<0.001	

Model 1: no adjustment for confounding factors. Model 2: adjusted for gender, age, ethnicity, marital status, average monthly income, and educational level. Model 3: adjusted for gender, age, ethnicity, marital status, average monthly income, educational level, length of service, smoking status, drinking status, leisure-time physical activity, DASH score, family history of hypertension, and family history of diabetes.

### Analysis of the association between shift work pattern and the incidence of CKM syndrome

According to Table 4, after adjusting for all confounding factors, the risk of CKM syndrome among workers on two shifts (RR = 1.677 95% CI: 1.391–2.022), three shifts (RR = 1.502, 95% CI: 1.223–1.844), and four shifts (RR = 1.529, 95% CI: 1.235–1.892) schedules was significantly higher than that of workers on fixed day shifts and the differences were statistically significant.

**Table 4:** Association between different shift work patterns and the incidence of CKM syndrome.

Shift work pattern	Case/total (incidence)	Model 1		Model 2		Model 3	
		RR (95% CI)	*p*	RR (95% CI)	*p*	RR (95% CI)	*p*
Fixed day shift	118/281 (42.0)	1.000	—	1.000	—	1.000	—
Two shifts	99/160 (61.9)	1.473 (1.226–1.770)	<0.001	1.469 (1.225–1.762)	<0.001	1.677 (1.391–2.022)	<0.001
Three shifts	84/141 (59.6)	1.419 (1.169–1.721)	<0.001	1.394 (1.151–1.688)	0.001	1.502 (1.223–1.844)	<0.001
Four shifts	63/108 (58.3)	1.389 (1.125–1.715)	0.002	1.469 (1.225–1.762)	0.002	1.529 (1.235–1.892)	<0.001

Model 1: no adjustment for confounding factors. Model 2: adjusted for gender, age, ethnicity, marital status, average monthly income, and educational level. Model 3: adjusted for gender, age, ethnicity, marital status, average monthly income, educational level, length of service, smoking status, drinking status, leisure-time physical activity, DASH score, family history of hypertension, and family history of diabetes.

### Analysis of the association between occupational physical activity and the incidence of CKM syndrome

Table 5 shows that after adjusting for all confounding factors, compared with workers with light or moderate OPA, those with heavy or very heavy OPA had an increased risk of CKM syndrome (RR = 1.432, 95% CI: 1.239–1.654), stage 1 CKM syndrome (RR = 1.778, 95% CI: 1.334–2.370), and stages 2–4 CKM syndrome (RR = 1.516, 95% CI: 1.244–1.848).

**Table 5:** Association between occupational physical activity and the incidence of CKM syndrome.

OPA	Case/total (incidence)	Model 1		Model 2		Model 3	
		RR (95% CI)	*p*	RR (95% CI)	*p*	RR (95% CI)	*p*
CKM syndrome							
Light or moderate	217/456 (47.6)	1.000	—	1.000	—	1.000	—
Heavy or very heavy	147/234 (62.8)	1.320 (1.150–1.515)	<0.001	1.424 (1.235–1.641)	<0.001	1.432 (1.239–1.654)	<0.001
CKM syndrome stage 1							
Light or moderate	73/456 (16.0)	1.000	—	1.000	—	1.000	
Heavy or very heavy	55/234 (23.5)	1.655 (1.241–2.209)	0.001	1.754 (1.320–2.332)	<0.001	1.778 (1.334–2.370)	<0.001
CKM syndrome stages 2–4							
Light or moderate	144/456 (31.5)	1.000	—	1.000		1.000	
Heavy or very heavy	92/234 (39.3)	1.367 (1.128–1.657)	0.001	1.512 (1.244–1.837)	<0.001	1.516 (1.244–1.848)	<0.001

Model 1: no adjustment for confounding factors. Model 2: adjusted for gender, age, ethnicity, marital status, average monthly income, and educational level. Model 3: adjusted for gender, age, ethnicity, marital status, average monthly income, educational level, length of service, smoking status, drinking status, leisure-time physical activity, DASH score, family history of hypertension, and family history of diabetes.

### Joint associations of shift work and OPA with CKM syndrome risk

As shown in Table 6, after adjusting for all confounding factors, the shift work plus heavy or very heavy OPA group had an increased risk of CKM syndrome compared with the reference group (RR = 2.193, 95% CI: 1.771–2.716).

**Table 6:** Joint associations of shift work and OPA with CKM syndrome risk.

Shift work	OPA	Case/total (incidence)	Model 1		Model 2		Model 3	
			RR (95% CI)	*p*	RR (95% CI)	*p*	RR (95% CI)	*p*
Fixed day shift	Light or moderate	69/180 (38.3)	1.000		1.000		1.000	
Shift work	Light or moderate	148/276 (53.6)	1.399 (1.128–1.735)	0.002	1.407 (1.140–1.735)	0.001	1.582 (1.276–1.962)	<0.001
Fixed day shift	Heavy or very heavy	49/101 (48.5)	1.266 (0.963–1.663)	0.091	1.404 (1.068–1.844)	0.015	1.464 (1.115–1.921)	0.006
Shift work	Heavy or very heavy	98/133 (73.7)	1.922 (1.556–2.374)	<0.001	2.015 (1.632–2.488)	<0.001	2.193 (1.771–2.716)	<0.001

Model 1: no adjustment for confounding factors. Model 2: Adjusted for gender, age, ethnicity, marital status, average monthly income, and educational level. Model 3: Adjusted for gender, age, ethnicity, marital status, average monthly income, educational level, length of service, smoking status, drinking status, leisure-time physical activity, DASH score, family history of hypertension, and family history of diabetes.

## Discussion

This study found that compared with workers on fixed day shifts, shift work and various shift patterns increased the risk of CKM syndrome, and the risk elevated with an increasing number of night shifts. Compared with workers engaged in light or moderate OPA, those involved in heavy and very heavy OPA have an increased risk of developing CKM syndrome. Combined exposure to shift work and heavy/very heavy OPA is associated with a higher risk of CKM syndrome.

Our findings show that shift work is a risk factor for CKM syndrome. Accumulating evidence has shown that shift work is associated with all components of CKM syndrome. Night-shift work is independently linked to an increased risk of obesity (35). Beyond obesity, multiple studies have confirmed that rotating shift work exacerbates insulin resistance and dyslipidemia, thereby elevating the risk of metabolic syndrome (36, 37). Regarding renal health, long-term night-shift exposure is closely associated with reduced glomerular filtration rate and albuminuria, suggesting its role in promoting early chronic kidney disease progression (38, 39). For cardiovascular outcomes, shift workers exhibit a higher incidence of hypertension, coronary heart disease and stroke, which is consistent with the elevated risk of the cardiovascular component of CKM syndrome (40–42). Collectively, studies across different populations support that shift work affects individual CKM components; the present study further extends this evidence to the overall spectrum of CKM syndrome, which is consistent with the findings of existing research. All shift patterns significantly increase workers’ CKM syndrome risk, with the two shifts having the most pronounced effect-its longer working hours and higher night shift frequency exert greater metabolic impacts (43). Additionally, we found a dose–response relationship between cumulative night shifts and CKM syndrome; previous studies on the dose–response relationship between night shifts and cardiovascular disease as well as metabolic syndrome also provide supporting evidence (44, 45). A United Kingdom Biobank cohort of over 96,000 participants reported a graded rise in CKM-related disease risk with longer, more intense, and cumulative night-shift exposure. Notably, they defined CKM as stroke, ischemic heart disease, heart failure, chronic kidney disease, or type 2 diabetes, while we used the AHA’s standardized CKM syndrome definition (14). The key physiological mechanism underlying these associations is chronic circadian disruption induced by long-term night and rotating shifts, which disturbs sleep–wake cycles, feeding rhythms, and hormonal oscillations (e.g., melatonin suppression, dysregulated cortisol and insulin rhythms) (46, 47). This circadian misalignment directly triggers insulin resistance, obesity, hypertension, and renal hemodynamic impairment, which collectively drive the development of CKM syndrome (48, 49). Our findings highlight the importance of monitoring the health of shift workers, especially those with long-term night shift exposure.

Studies have shown that heavy and very heavy OPA is a risk factor for CKM syndrome. A Danish study found that heavy OPA elevates the risk of cardiovascular events (50). OPA is usually continuous and repetitive, and engaging in heavy occupational physical activity may cause a sharp increase in blood pressure (51, 52). In addition, a study by Sorensen et al. (53) found that exposure to heavy physical activity can lead to impaired renal function. Coenen et al. (54) found that men with high-level occupational physical activity had an 18% higher risk of early mortality compared with those with low-level occupational physical activity. Similarly, Jordakieva et al. (25) demonstrated that high-intensity OPA was associated with cardiovascular disease mortality. These findings collectively suggest that heavy OPA may serve as a risk factor for the development of CKM syndrome. However, studies on the association between occupational physical activity and metabolic diseases have yielded inconsistent conclusions. A United States cohort study found that high-intensity occupational physical activity is associated with an increased risk of diabetes (18). In contrast, a study by Kuwahara et al. (55) showed that higher occupational physical activity is associated with a lower risk of metabolic syndrome. Hu et al. (20) reported that moderate-to-high occupational physical activity independently and significantly reduced the risk of type 2 diabetes in middle-aged general populations. Both studies assessed OPA levels based on participants’ self-reported physical activity, which differs from the objective workload classification used in the present study. In our study, the intensity of the OPA was determined in accordance with studies on the classification of physical work intensity for petroleum fieldwork. This may account for the inconsistent findings across studies.

This study found that compared with workers without shift work and with light or moderate OPA, those with shift work and heavy or very heavy OPA had a higher incidence of CKM syndrome. Individuals engaged in physical work are more likely to work frequent shifts (56). Both shift work and high-intensity physical work can stimulate cortisol release; under their combined effect, cortisol remains at a high level for a long time, which may induce anxiety, increase blood pressure, and disrupt metabolic function (57). The cardiovascular system has extremely low tolerance to “rhythm disturbance + stress load”, and irreversible damage is prone to occur under dual pressure. The blood pressure of normal people shows a circadian rhythm of “higher during the day and lower at night”, which is disrupted by shift work, leading to increased rather than decreased blood pressure at night. Additionally, the stress response caused by high-intensity work further activates the sympathetic nervous system, keeping blood pressure at a persistently high level, which may lead to essential hypertension in the long term (58, 59). These findings confirm that while shift work and heavy OPA independently increase the risk of CKM syndrome, they also promote each other and jointly increase the risk of CKM syndrome. Therefore, enterprises should closely monitor the health status of workers who perform both shift work and heavy occupational physical activity, and implement targeted job adjustments as needed.

The main strength of this study is its first exploration of the association between shift work, OPA and CKM syndrome, as well as the impact of their interaction on CKM syndrome risk. However, it has several limitations: (1) Subjects were from the petroleum and petrochemical industry with specific occupational exposures; future studies should expand the sample size and include more confounding factors. (2) Only baseline data were used; follow-up variable changes may have biased results. Future dynamic monitoring can improve study reliability. (3) This study has certain limitations. Objective circadian rhythm biomarkers such as melatonin and cortisol were not measured, which could be incorporated in future studies.

## Conclusion

This study demonstrated that all shift patterns are associated with an increased risk of CKM syndrome, and a clear dose–response relationship exists between cumulative night shift exposure and CKM risk. Heavy/very OPA also independently elevates CKM risk. Workers with combined exposure to shift work and heavy/very heavy OPA face the highest risk. To reduce CKM incidence, enhanced health protection is recommended for these vulnerable groups. Further large-sample prospective and mechanistic studies are warranted to validate these findings.

## Data Availability

The raw data supporting the conclusions of this article will be made available by the authors, without undue reservation.

## Glossary

AHA: American Heart Association
CKM: Cardiovascular-Kidney-Metabolic
CKD: chronic kidney disease
CVD: cardiovascular diseases
BMI: body mass index
TG: triglyceride
SBP: systolic blood pressure
DPB: diastolic blood pressure
HbA1c: glycated hemoglobin A1c
TC: total cholesterol
HDL-C: high-density lipoprotein cholesterol
GFR: glomerular filtration rate
RERI: relative excess risk due to interaction
AP: attributable proportion
CI: confidence interval
S: synergy index
SD: standard deviations
RR: relative risk
OHSPIW: occupational health study of petroleum industry workers
ILO: International Labour Organization
DASH: dietary approaches to stop hypertension
