# Supplementary material for: The association between shift work, occupational physical activity and cardiovascular-kidney-metabolic syndrome: a cohort study in China
Source: Front Public Health. 2026 Jun 3;14:1831468. doi: 10.3389/fpubh.2026.1831468 (PMC13272170; doi:10.3389/fpubh.2026.1831468)
Supplement: Supplementary file 1 [file Table_1.docx]

Table S1 Baseline Characteristics of Included and Excluded Participants

| Variable | Inclusion N=690 (n, %) | Exclusion N=110 (n, %) | χ^2^ | *P* |
| --- | --- | --- | --- | --- |
| Gender |  |  | 0.015 | 0.904 |
| Male | 347(50.3) | 56(50.9) |  |  |
| Female | 343(49.7) | 54(49.1) |  |  |
| Age (years) |  |  | 0.007 | 0.997 |
| ≤39 | 212(30.7) | 34(30.9) |  |  |
| 40-49 | 304(44.1) | 48(43.6) |  |  |
| ≥50 | 174(25.2) | 28(25.5) |  |  |
| Ethnicity |  |  | 0.057 | 0.811 |
| Han nationality | 662(95.9) | 105(95.5) |  |  |
| Other ethnicities | 28(4.1) | 5((4.5) |  |  |
| Marital status |  |  | 0.138 | 0.911 |
| Unmarried | 31(4.5) | 5(4.5) |  |  |
| Married | 615(89.1) | 99(90.0) |  |  |
| Other | 44(6.4) | 6(5.5) |  |  |
| Educational level |  |  | 23.236 | <0.001 |
| Senior high school or below | 89(12.9)_ | 33(30.0) |  |  |
| Junior college, | 527(76.5) | 63(57.3) |  |  |
| Bachelor's degree or above | 74(10.7) | 14(20.3) |  |  |
| Average monthly income (yuan) |  |  | 5.191 | 0.075 |
| ≤2999 | 19(2.8) | 4(3.6) |  |  |
| 3000-4999 | 615(89.1) | 90(81.8) |  |  |
| ≥5000 | 56(8.1) | 16(14.5) |  |  |
| Length of service (years) |  |  | 0.351 | 0.839 |
| ≤9 | 82(11.9) | 11(10.0) |  |  |
| 10-19 | 195(28.3) | 31(28.2) |  |  |
| ≥20 | 413(59.9) | 68(61.8) |  |  |
| Smoking status |  |  | 10.332 | 0.016 |
| Non-smoking | 518(75.2) | 79(71.8) |  |  |
| Regular smoking | 62(9.0) | 20(18.2) |  |  |
| Occasional smoking | 83(12.0) | 9(8.2) |  |  |
| Quit smoking | 27(3.9) | 2(1.9) |  |  |
| Drinking status |  |  | 0.724 | 0.867 |
| Non-drinking | 331(48.0) | 49(44.5) |  |  |
| Regular drinking | 193(28.0) | 33(30.0) |  |  |
| Occasional drinking | 158(23.9) | 26(23.6) |  |  |
| Quit-drinking | 8(1.2) | 2(1.8) |  |  |
| Leisure-time physical activity |  |  | 1.800 | 0.615 |
| no physical activity" | 79(11.5) | 10(9.1) |  |  |
| <3 times/week | 269(39.0) | 50(45.5) |  |  |
| ≥3 times/week | 115(16.7) | 17(15.5) |  |  |
| irregular physical activity" | 227(32.9) | 33(30.0) |  |  |
| DASH score (points) |  |  | 0.868 | 0.648 |
| ≤22 | 188(27.2) | 28(25.5) |  |  |
| 23-26 | 282(40.9) | 42(38.2) |  |  |
| ≥27 | 220(31.9) | 40(36.4) |  |  |
| Family history of hypertension |  |  | 0.227 | 0.633 |
| No | 553(80.1) | 86(78.2) |  |  |
| Yes | 137(19.9) | 24(21.8) |  |  |
| Family history of diabetes |  |  |  |  |
| No | 437(63.3) | 65(59.1) | 0.731 | 0.393 |
| Yes | 253(36.7) | 45(40.9) |  |  |
| Shift work |  |  |  |  |
| No | 281(40.7) | 45(40.9) | 0.001 | 0.971 |
| Yes | 409(59.3) | 65(59.1) |  |  |
| OPA |  |  |  |  |
| Light or moderate | 456(66.1) | 75((68.2) | 0.187 | 0.666 |
| Heavy or very heavy | 234(33.9) | 35(31.8) |  |  |
